# The resistance of peanut to soil-borne pathogens improved by rhizosphere probiotics under calcium treatment

**DOI:** 10.1186/s12866-021-02355-3

**Published:** 2021-10-29

**Authors:** Wei Zhang, Bo-wen Zhang, Jie-fu Deng, Lin Li, Tu-yong Yi, Yan-yun Hong

**Affiliations:** 1 Hunan Provincial Key Laboratory for Biology and Control of Plant Pests, College of Plant Protection, Hunan Agricultural University, Changsha, China; 2 Research Centre for Hunan Peanut Engineering Technology, College of Agriculture, Hunan Agricultural University, Changsha, China

**Keywords:** Peanut, Calcium, Rhizosphere, Soil-borne pathogens, Resistance

## Abstract

**Background:**

Peanut (*Arachis hypogaea* L.) is an important oil and economic crop. Calcium modulates plants in response to abiotic stresses and improves plant resistance to pathogens. Enrichment of beneficial microorganisms in the rhizosphere is associated with plant disease resistance and soil development. The purpose of this study was to analyze the differences in peanut rhizosphere microbial community structure between the calcium treatment and the control during two growth stages and to explain why calcium application could improve the resistance of peanuts to soil-borne pathogens.

**Results:**

The 16S rDNA amplicon sequencing of rhizosphere microbiome showed that calcium application significantly enriched *Serratia marcescens* and other three dominant strains at the seedling stage. At the pod filling stage, ten dominant stains such as *Sphingomonas changbaiensis* and *Novosphingobium panipatense* were enriched by calcium. *Serratia marcescens* aseptic fermentation filtrate was mixed with PDA medium and inoculated with the main soil-borne pathogens in the seedling stage, which could inhibit the growth of *Fusarium solani* and *Aspergillus flavus*. The aseptic fermentation filtrate of *Novosphingobium panipatense* was mixed with PDA medium and inoculated with the main soil-borne pathogens in the pod filling stage, which could inhibit the growth of *Sclerotium rolfsii* and *Leptosphaerulina arachidicola*.

**Conclusions:**

Calcium application increases the resistance of peanuts to soil-borne pathogens by enriching them with specific dominant bacteria.

**Supplementary Information:**

The online version contains supplementary material available at 10.1186/s12866-021-02355-3.

## Background

Peanuts were widely cultivated as important oil and economic crops [1]. Many factors affect the peanut production. Blind fertilization and continuous cropping have led to the accumulation of toxic substances and the epidemic of severe diseases, which have become the main constraints to peanut production [2, 3]. Soil is not only the medium for peanut growth, but also the main supplier of the nutrients it needs. The abundance of soil nutrients is directly related to the yield and quality of peanuts [4]. Rational fertilization is an important guarantee for regulating soil nutrient status, balancing peanut’s nutrient demand, and improving yield and quality [5].

Peanut is one of the calciphilous plants. Soil calcium deficiency would cause a significant reduction in peanut yield and quality [6]. Calcium deficiency at the seedling stage could cause leaf chlorosis, broken petioles, wilting and death in severe cases, non-differentiated roots, weak roots with few nodules etc. Calcium deficiency at the pod filling stage would cause the reduction of pod development, hollow kernels and easily rotten fruits [7, 8]. The empty shell rate increased significantly, resulting in severe yield reduction [9]. Calcium deficiency could even induce physiological lesions in the seeds and make them more susceptible to fungal infection. Calcium plays an irreplaceable role in promoting growth and ensuring the high yield and quality of peanuts [10, 11].

Continuous cropping and selective nutrient absorption made the soil-borne pathogens such as root rot, southern blight, crown rot, as well as many pathogens of foliar diseases, remain in the soil [12]. *Fusarium* infestation of peanuts generally causes root rot which results in browning and rotting of the roots and crumpling and dry rot of the taproots. It could occur at all growth stages and could be aggravated by heavy rainfall [13]. *Sclerotium rolfsii* Sacc. usually cause southern blight, which could form white filamentous hyphae at the the site of infestation with the sclerotia formed in the later growth stage. Subsequently, the leaves turned yellow or brown to wither, and then the plants wilted and died [14]. Peanut leaf spot diseases include the brown spot (*Cercospora arachidicola*), the black spot (*Cercosporidium personatum*), the scorch spot (*Leptosphaerulina arachidicola*), the web blotch (*Phoma arachidicola*) and so on. In the middle and late stages of growth, mixed infestations of multiple pathogens caused a mixture of symptoms, mainly destroying chlorophyll and reducing the photosynthetic rate. Defoliation caused by a large number of spots could severely reduce dry matter accumulation and impair pod maturation, which could lead to large yield reductions [15].

Researchers have carried out a series of relevant projects to address soil nutrient imbalances, soil-borne disease epidemics, and severe yield reductions caused by improper field management [16]. Fruitfully progress has been made in the antagonistic screening of biocontrol bacteria and the development of fertilizers with reasonable nutrient ratios [17, 18]. It was also found that the microbiome could extend the plant’s defense ability [19, 20]. PGPR (Plant growth promoting rhizobacteria) could enhance host resistance to pathogens by inducing systemic resistance (ISR) in plants. Root exudates could also influence the structure of the rhizosphere microbial community by altering the physical and chemical properties of the soil to attract microorganisms from the nearby soil environment. Berendsen et al. [21] found that *Hyaloperonospora arabidopsidis* infecting the leaves of *Arabidopsis thaliana* promoted the proliferation of three specific ISR-inducing bacteria in the rhizosphere and that these protective bacteria appeared to persist in the soil and act as a downy mildew resistant agent for the plant. Wang et al. [22] demonstrated that alterations in the composition of peanut root exudates contributed to the enhancement of rhizobial nodulation mediated by the endophytic fungus *Phomopsis liquidambaris*. Yuan et al. [23] demonstrated that *A. thaliana* can strengthen its resistance to disease by modifying the composition of root exudates to regulate the structure of the rhizosphere microbial community after infestation by foliar pathogens. Furthermore, inoculation with microbial mixtures is more effective against diseases than with a single strain. Molina et al. [24] improved the resistance of *A. thaliana* to fungal pathogens by inoculation with *Pseudomonas putida*, *Sphingomonas* sp., *Acinetobacter* sp. and *Azospirillum brasilense*.

Ca acts as the second messenger and the essential nutrient for plants, could regulate plant growth and development. Cui et al. [25] found that the combination of arbuscular mycorrhizal fungi with exogenous calcium promoted the growth of peanut seedlings under continuous cropping. Chao et al. [26] treated Djulis sprouts with 5 mM calcium carbonate improved antioxidant capacity, reduced the content of reactive oxygen species, and promoted crop growth. When plants were threatened by external stimuli such as adverse chemical stress, salt stress, low-temperature stress, Ca^2+^ channel was opened and large amounts of free Ca^2+^ combined with CaM to activate the target enzyme and induce corresponding physiological and biochemical processes to resist external stress. Li et al. [27] indicated that exogenous calcium could effectively improve the low temperature tolerance of loquat fruits and promote the accumulation of endogenous osmolytes such as proline, GABA and PAS. Liu et al. [28] found that ANNEXIN1 as a calcium transporter could mediate cold-induced calcium signaling in plants and regulate the expression of cold resistance-related genes CBF and COR, as a way to enhance cold resistance in plants. Calcium regulated early-triggered systemic acquired resistance and contributed to plant defence response, therefore enhancing the host plant’s resistance to pathogens [29]. Bundó et al. [30] discovered that the OsCPK4 subtype as a member of the calcium-dependent protein kinase family, which was overexpressed in rice, could improve the resistance to rice blast. Wei et al. [31] found that the calcium-dependent protein kinase TACPK7-D could positively regulate wheat resistance to sharp eyespot.

The major studies on plant-microbe interactions always based on *A. thaliana* or model crops like rice and maize. However, little is known about interactions between the peanut rhizosphere microbial community and the plant. Studies on calcium signaling in plant immunity have also been conducted in *A. thaliana* to elucidate specific regulatory pathways. Field trials have found that calcium application enhanced peanut resistance to common pests and diseases, but its machine is unclear. In this study, the 16S rDNA V3/V4 region was sequenced in the rhizosphere microbiome of peanuts at different growth stages from the samples with and without calcium treatment. The dominant strains obtained from the screening were cultured in plate confrontations with the isolated soil-borne pathogens to verify their bacterial inhibition efficacy. The result would provide a preliminary analysis of the reasons why calcium could enhance peanut resistance. It provided a theoretical basis for the rational application of mineral elements, balancing soil nutrients, improving soil quality and reducing the incidence of soil-borne diseases of peanuts.

## Results

### Composition of Peanut rhizosphere microbial community

The pH of the soil was evaluated respectively at the pre-sowing stage, the seedling stage and the pod filling stage for the sample group with and without calcium treatment. The samples were taken from a depth of 20 cm. The initial pH of the soil was 4.613 ± 0.021 before sowing, 4.643 ± 0.012 at the seedling stage, and the pod filling stage was 4.776 ± 0.028 (Values are mean ± standard error of three replicates). The pH fluctuated less at the seedling stage and varied slightly more at the pod filling stage. Overall, the pH at different growth stages did not change obviously from pre-sowing.

Based on IonS5™XL sequencing platform, small fragment libraries were constructed for single-end sequencing. By shearing and filtering Reads, an average of 82,608 reads were measured per sample, and 77,872 valid data were obtained on average after QC (Quality Control), with a QC efficiency of 94.32%. The sequences were clustered with 97% identity and a total of 2378 OTUs (Operational taxonomic units) were obtained. By comparing the species annotation with the Silva132 database and counting the different taxonomic levels, it was found that the number of OTUs that could be annotated to the database was 2372 (99.75%) out of 2378 OTUs. The percentage of annotation to each taxonomic level was 99.75% for the kingdom, 94.62% for the phylum, 91.17% for the class, 83.22% for the order, 75.11% for the family, 51.77% for the genus, and 14.30% for the species. Samples were grouped as follows: S.S: The rhizosphere microbiome without calcium application at the seedling stage; SC.S: The rhizosphere microbiome with calcium application at the seedling stage; P.S: The rhizosphere microbiome without calcium application at the pod filling stage; PC.S: The rhizosphere microbiome with calcium application at the pod filling stage.

**Fig. 1:**
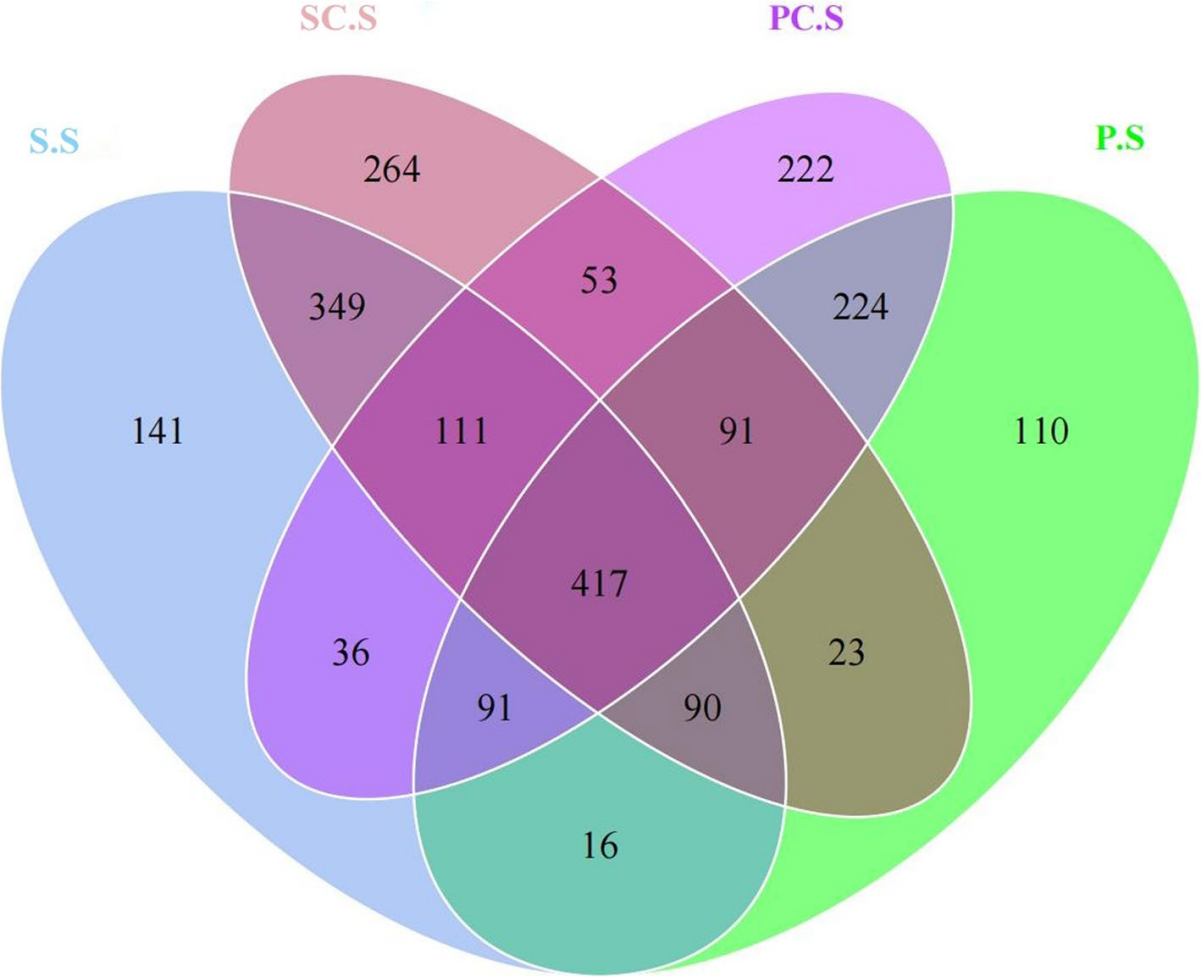
Venn diagram based on the OTUs of the rhizosphere microbiome. S.S: The rhizosphere microbiome without calcium application at the seedling stage; SC.S: The rhizosphere microbiome with calcium application at the seedling stage; P.S: The rhizosphere microbiome without calcium application at the pod filling stage; PC.S: The rhizosphere microbiome with calcium application at the pod filling stage

A total of 417 OTUs were shared in all samples at the seedling and pod filling stages. The number of unique OTUs in the groups with calcium was significantly higher than that of the groups without calcium. The number of unique OTUs in the non-calcium group at the seedling stage was 141, which was lower than the 264 unique OTUs in the group with calcium application. The 222 unique OTUs in the group with calcium were significantly higher than the 110 unique OTUs in the non-calcium group at the pod filling stage (Fig. 1).

### Analysis on differences of Peanut rhizosphere microbial communities between groups

**Fig. 2:**
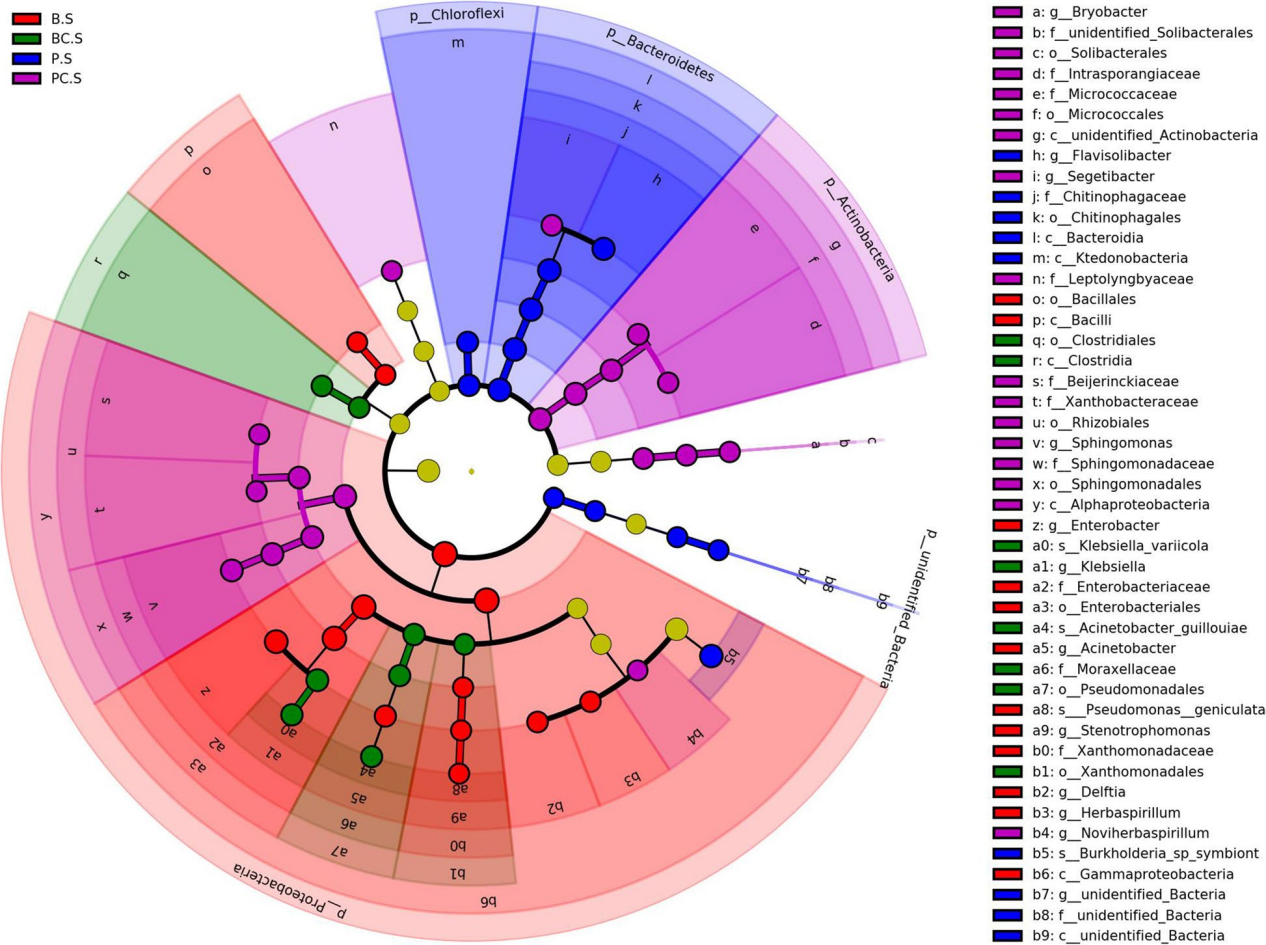
LEfSe analysis cladogram of the rhizosphere microbiome. The red nodes represented the dominant strains as important Biomarkers in the group without calcium application at the seedling stage; The green nodes represented the dominant strains as important Biomarkers in the group with calcium application at the seedling stage; The blue nodes represent the dominant strains as important Biomarkers in the group without calcium application at the pod filling stage; The purple nodes represent the dominant strains as important Biomarkers in the group with calcium application at the pod filling stage

LEfSe analysis (LDA Effect Size) was used to compare the significant differences in the abundance of peanut rhizosphere microbial communities between the samples with and without calcium application from different growth stages at the genus and species levels. The results were presented in a cladogram (Fig. 2). The important Biomarker in the rhizosphere microbial community without calcium at the seedling stage was *Pseudomonas geniculata.* The vital Biomarkers in the microbial community with calcium at the seedling stage were *Acinetobacter guillouiae* and *Klebsiella variicola*. The Biomarker in the microbial community without calcium at the pod filling stage was *Burkholderia* sp*. symbiont*. However, the Biomarkers for the microbial community with calcium at the pod filling stage has not been identified at the level of species. At the taxonomic level of genus, *Bryobacter*, *Segetibacter*, *Sphingomonas* and *Noviherbaspirillum* as the dominant genera were biologically valuable in the rhizosphere microbial community at the pod filling stage under calcium treatment.

**Fig. 3:**
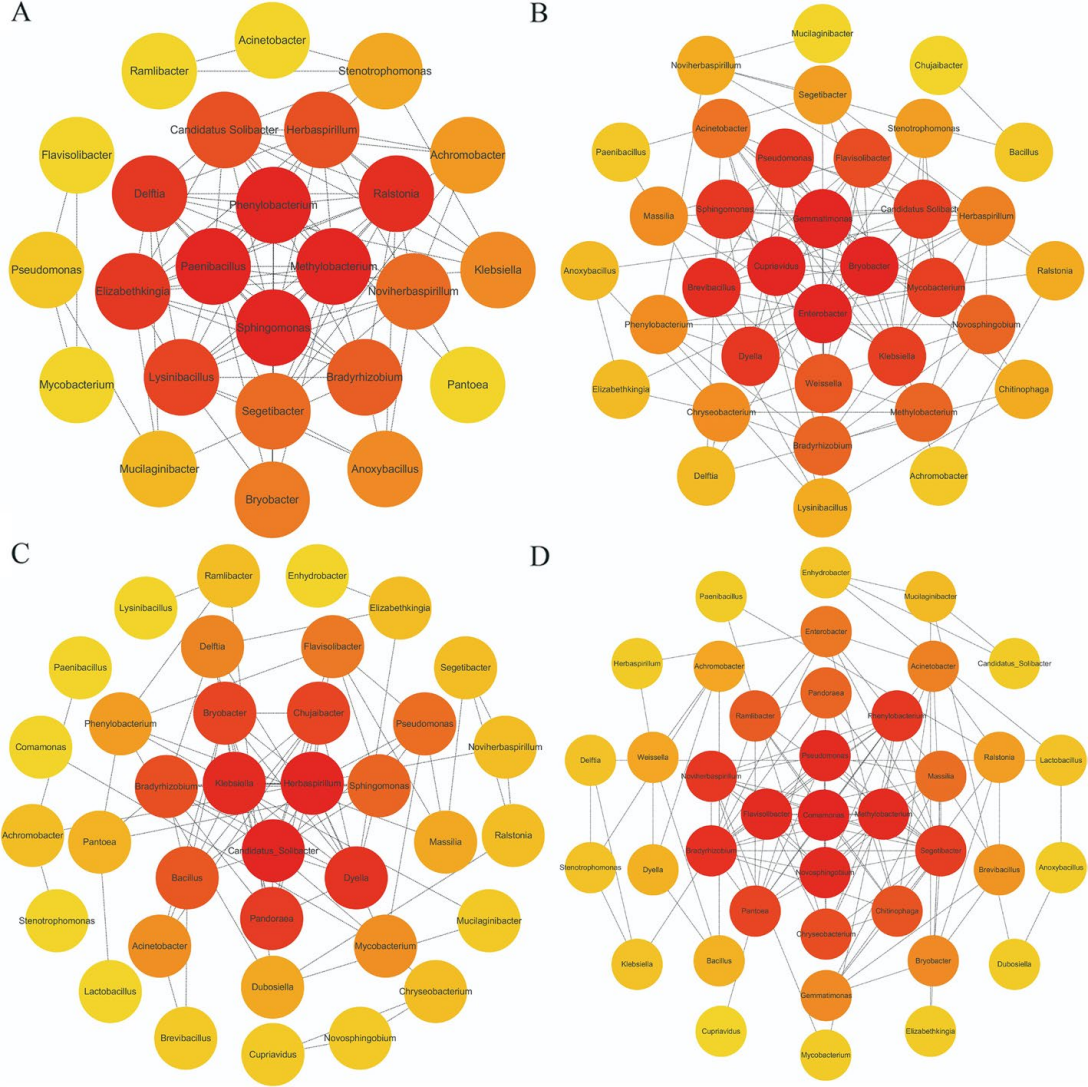
The interaction network diagrams of the dominant genera with relative abundance of Top50. **A** The interaction network diagram of dominant genera with relative abundance of Top50 in the microbial community without calcium. *Phenylobacterium*, *Methylobacterium*, *Sphingomonas*, *Paenibacillus* were the main core bacteria in the group. **B** The interaction network diagram of dominant genera with relative abundance of Top50 in the microbial community with calcium. *Cupriavidus*, *Gemmatimonas*, *Bryobacter*, *Enterobacter* were the main core bacteria in the group. **C** The interaction network diagram of dominant genera with relative abundance of Top50 in the microbial community at the seedling stage. *Candidatus Solibacter* and *Herbaspirillum* were the main core bacteria in the group. **D** The interaction network diagram of dominant genera with relative abundance of Top50 in the microbial community at the pod filling stage. *Comamonas*, *Novosphingobium*, *Methylobacterium*, *Pseudomonas*, and *Flavisolibacter* were the main core bacteria in the group

The genus correlation of the rhizosphere microbial community at different growth stages from the calcium-applied group and the control could be visualized display by the interaction network diagram (Fig. 3). The number of key nodes and effective connections between the dominant genera in the calcium-applied group was higher than those in the control. Meanwhile, the number of key nodes and effective connections between the dominant genera in the pod filling stage was slightly higher than those in the seedling stage. The MCC value of core genera from each group was calculated and ranked according to the MCC algorithm of the cytohubba’s plug-in (Subtab. 1). The result showed that the core genera in the microbial community without calcium were *Phenylobacterium, Methylobacterium, Sphingomonas* and *Paenibacillus* (MCC = 85,800). Core genera in the rhizosphere microbial community with calcium were *Cupriavidus, Gemmatimonas, Bryobacter* and *Enterobacter* (MCC = 6480). The core genera of the microbial community at the seedling stage were *Candidatus Solibacter* and *Herbaspirillum* (MCC = 1134)*.* Core genera of the microbial community at the pod filling stage was *Comamonas* (MCC = 81,528)*. Bryobacter* is the core genus shared by the calcium-applied group at the seedling stage. However, *Pseudomonas* is the core genus shared by the calcium-applied group at the pod filling stage.

**Fig. 4:**
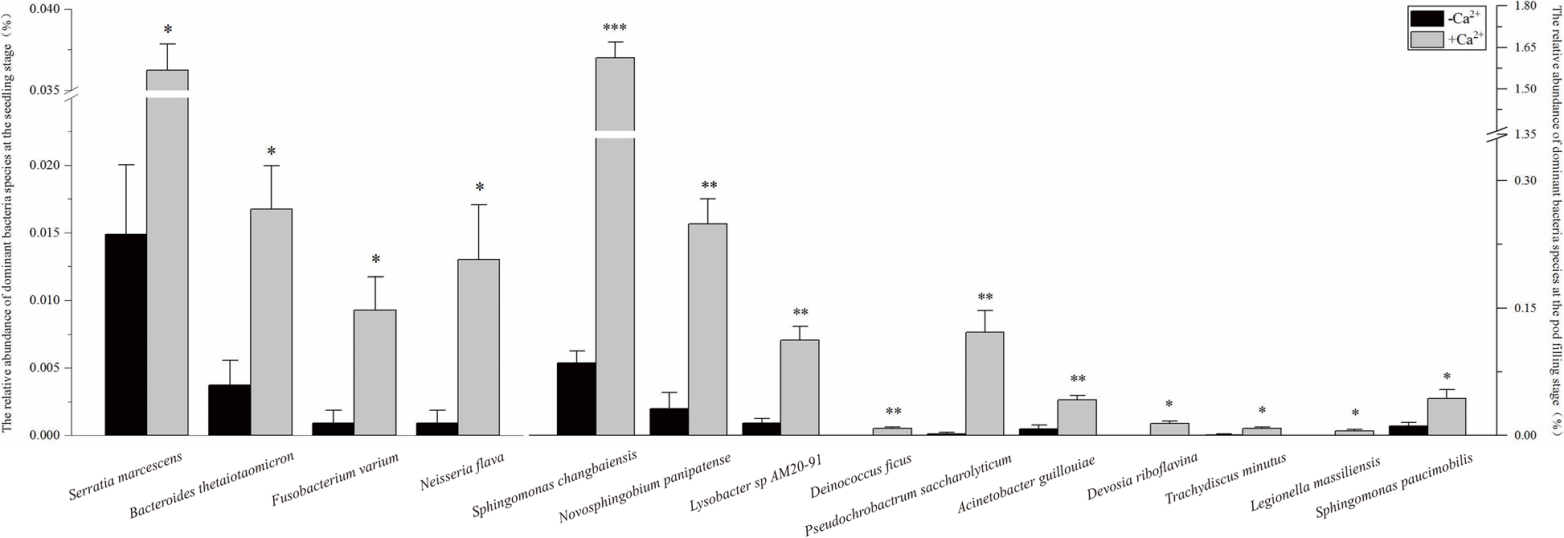
Histogram of the t-test for the relative abundance of all species of rhizosphere microbiome. *Serratia marcescens, Bacteroides thetaiotaomicron, Fusobacterium varium* and *Neisseria flava* were the dominant strains which had significant difference between the group with and without calcium treatment at the seedling stage (In descending order of *p* value), the other strains were the dominant species in the rhizosphere microbial community which had significant difference between the group with and without calcium treatment at the pod filling stage((*p < 0.05, **p < 0.01, ***p < 0.001)

The results of t-test showed that: *Serratia marcescens* with other three strains were enriched under calcium treatment at the seedling stage (*p < 0.05). *Sphingomonas changbaiensis* and *Novosphingobium panipatense* with other eight strains were significantly enriched under calcium treatment at the pod filling stage. Among them, *Sphingomonas changbaiensis* were extremely significantly enriched by calcium application (***p < 0.001), *Novosphingobium panipatense, Lysobacter* sp.AM20–91*, Pseudochrobactrum saccharolyticum, Deinococcus ficus* and *Acinetobacter guillouiae* were significantly enriched by calcium (**p < 0.01). The relative abundance of *Devosia riboflavina, Trachydiscus minutus, Legionella massiliensis* and *Sphingomonas paucimobilis* were different between calcium treatment and control groups at the pod filling stage (Fig. 4, Tables 1 and 2).

**Table 1:** T-test for the relative abundance of dominantspecies at the seedling stage

Taxonomy	Relative abundance (%)		*t*	*P*
	S.S	SC.S	0.141	
*Serratia marcescens*	0.0149 ± 0.0052	0.0362 ± 0.0016	−3.944	0.017
*Bacteroides thetaiotaomicron*	0.0037 ± 0.0019	0.0167 ± 0.0032	−3.500	0.025
*Fusobacterium varium*	0.0009 ± 0.0009	0.0093 ± 0.0025	−3.182	0.033
*Neisseria flava*	0.0009 ± 0.0009	0.0130 ± 0.0041	−2.907	0.044

Note: Values are mean ± standard error of three replicates. S.S: Rhizosphere soil microbiome without calcium application at the seedling stage. SC.S: Rhizosphere soil microbiome with calcium application at the seedling stage

**Table 2:** T-test for the relative abundance of dominantspecies at the pod filling stage

Taxonomy	Relative abundance (%)		*t*	*P*
	P.S	PC.S	0.141	
*Sphingomonas changbaiensis*	0.0855 ± 0.0140	1.6120 ± 0.0575	−8.531	0.001
*Novosphingobium panipatense*	0.0316 ± 0.0191	0.2490 ± 0.2490	−6.234	0.003
*Lysobacter* sp. AM20–91	0.0149 ± 0.0049	0.1124 ± 0.0162	−5.763	0.004
*Deinococcus ficus*	0	0.0084 ± 0.0016	−5.196	0.007
*Pseudochrobactrum saccharolyticum*	0.0019 ± 0.0019	0.1217 ± 0.0255	−4.695	0.009
*Acinetobacter guillouiae*	0.0074 ± 0.0049	0.0418 ± 0.0056	−4.625	0.010
*Devosia riboflavina*	0	0.0139 ± 0.0032	−4.330	0.012
*Trachydiscus minutus*	0.0009 ± 0.0009	0.0084 ± 0.0016	−4.000	0.016
*Legionella massiliensis*	0	0.0056 ± 0.0016	−3.464	0.026
*Sphingomonas paucimobilis*	0.0112 ± 0.0043	0.0437 ± 0.0043	−2.877	0.045

Note: Values are mean ± standard error of three replicates. P.S: Rhizosphere soil microbiome without calcium application at the pod filling stage. PC.S: Rhizosphere soil microbiome with calcium application at the pod filling stage

### Inhibition of Main pathogens by dominant calcium-enriched strains atDifferent growth stages

**Fig. 5:**
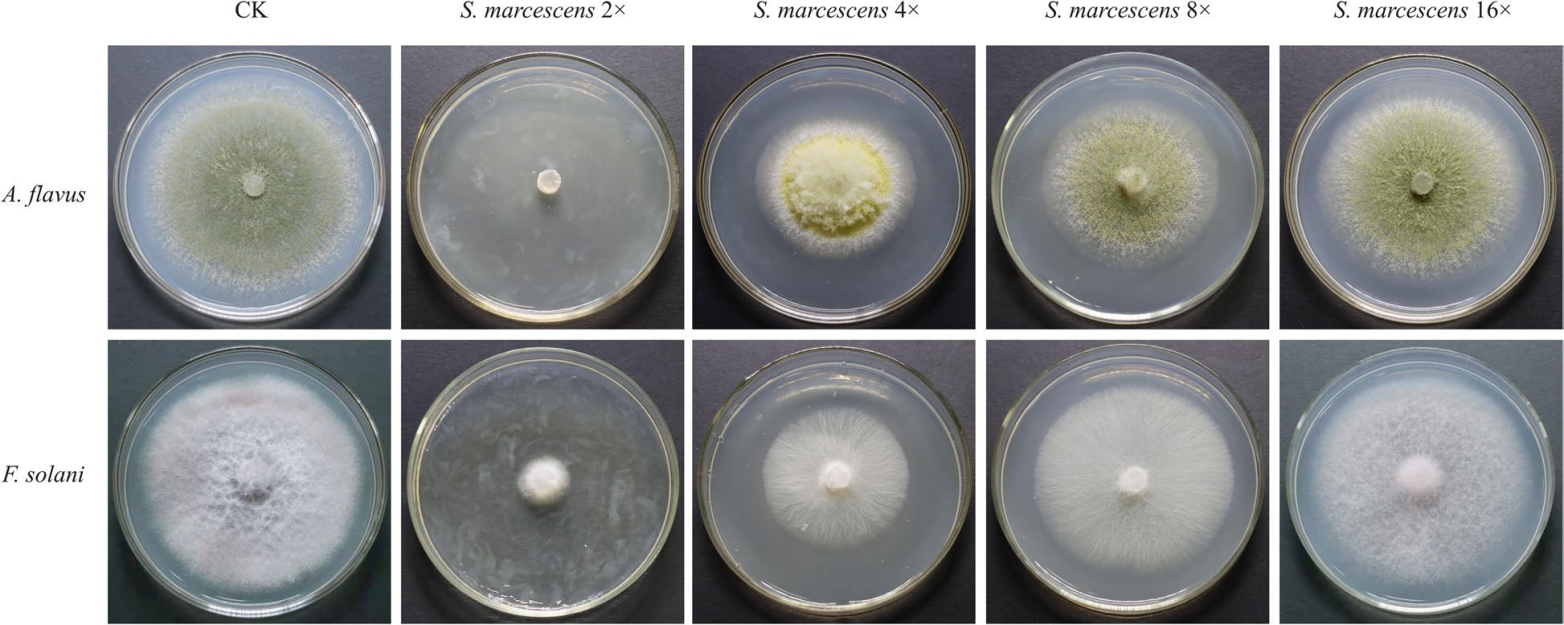
*S. marcescens* confronts the main pathogens *F. solani* and *A.flavus. S. marcescens* 2 × (50% fermentation filtrate mixed with 50% PDA nutrient medium); *S. marcescens* 4 × (25% fermentation filtrate mixed with 75% PDA nutrient medium); *S. marcescens* 8 × (12.5% fermentation filtrate mixed with 87.5% PDA nutrient medium); *S. marcescens* 16 × (6.25% fermentation filtrate mixed with 93.75% PDA nutrient medium). The main pathogens *F. solani* and *A. flavus* were inoculated on the mixed PDA plate for antagonistic confrontation culture for 7 days

**Fig. 6:**
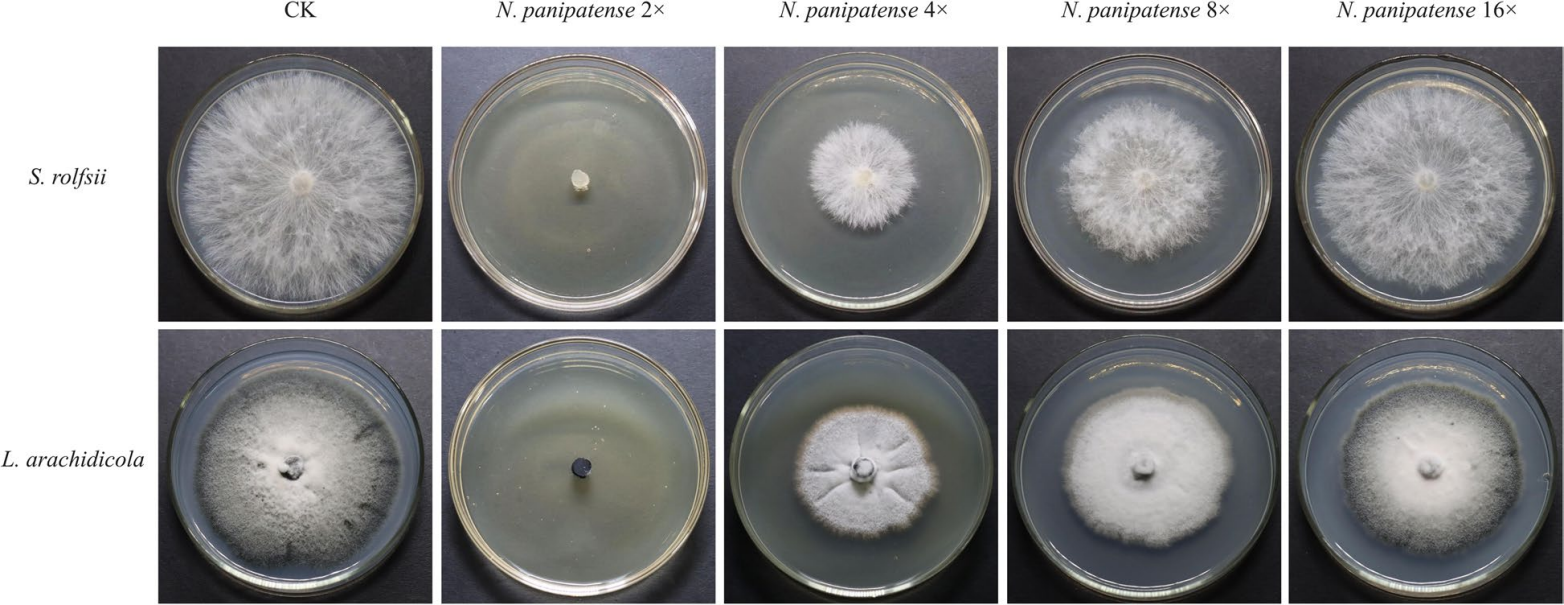
*N. panipatense* confronts the main pathogens *S. rolfsii* and *L. arachidicola*. *N. panipatense* 2 × (50% fermentation filtrate mixed with 50% PDA nutrient medium); *N. panipatense* 4 × (25% fermentation filtrate mixed with 75% PDA nutrient medium); *N. panipatense* 8 × (12.5% fermentation filtrate mixed with 87.5% PDA nutrient medium); *N. panipatense* 16 × (6.25% fermentation filtrate mixed with 93.75% PDA nutrient medium). The main pathogens *S. rolfsii* and *L. arachidicola* were inoculated on the mixed PDA plate for antagonistic confrontation culture for 7 days

Significantly enriched strains *Serratia marcescens* at the seedling stage and *Novosphingobium panipatense* at the pod filling stage were inoculated with the soil-borne pathogens (Seedling stage: *Fusarium solani* and *Aspergillus flavus*; Pod filling stage: *Sclerotium rolfsii* and *Leptosphaerulina arachidicola*.) isolated from the infected plants. The inhibition rate was calculated by the linear regression analysis. The results confirmed that *S. marcescens* and *N. panipatense* could significantly suppress the main soil-borne diseases at the same stage (Subtab. 2). The 2-fold dilution of the fermentation filtrates had the strongest inhibitory effect on the pathogens isolated. The inhibition rate of *F. solani* and *A. flavus* by the fermentation filtrate of *S. marcescens* were 82.7 and 88.8%. Meanwhile, the inhibition rate of *S. rolfsii* and *L. arachidicola* by 2-fold dilution fermentation filtrate of *N. panipatense* were 91.9 and 90.78% (Figs. 5 and 6). Gradient dilution of *S. marcescens* fermentation filtrate exhibited better inhibition of *A. flavus*, with a higher correlation coefficient between dilution ratios and inhibition rates (Subfig. 1). Meanwhile, gradient dilution of *N. panipatense* fermentation filtrate exhibited better inhibition of *S. rolfsii*, with a higher correlation coefficient between dilution ratios and inhibition rates.

## Discussion

Peanut is one of the calciphilous plants. Calcium deficiency could affect the development of pods, reduce the rate of seed kernel fullness, resulting in a substantial reduction in the yield and quality. Most researches focused on the molecular mechanism of calcium affecting pod development. Chen et al. [32] identified several key miRNAs along with target genes that regulate embryo abortion under the condition of calcium deficiency through transcriptome analysis. However, Yang et al. [33] suggested that in the early stage of peanut pod development, calcium preferentially promotes nutrient absorption and shell development but had little effect on the seed kernel formation. In this study, a survey of common diseases on peanuts cultivated in Yunyuan practice base found that the main diseases easily infected at different stages were not the same. The main diseases at the seedling stage were mainly root rot, with very few plants infected with southern blight. Infection with brown spots started at the acicula forming stage, and lasted until the pod filling stage. Brown spots, black spots, and scorch spots were mixed throughout the pod maturing stage, with scorch spots being more severe. Previous studies showed that calcium application could reduce the disease index of leaf spots and improve the resistance to related diseases. Therefore, 16S rDNA amplicon sequencing was performed on the peanut rhizosphere microbiome at different growth stages, which were treated with or without calcium, to further investigate the mechanism associated with calcium application to improve disease resistance in peanuts.

Infection with root rot is one of the main causes of yield reduction in peanuts. Continuous cropping and frequent use with the increasing dosages of pesticides made the pathogen’s resistance to pesticides increase each passing year, thus the incidence of the disease increased exponentially. The use of antagonistic microorganisms to control root rot has become a current research trend. Sun et al. [34] found that pre-inoculation with the root endophyte *Phomopsis liquidambaris* B3 could induce the resistance to *Fusarium oxysporum* by activating the salicylic acid-dependent signaling pathway. Guo et al. [35] demonstrated that *Serratia marcescens* significantly inhibited spore germination and mycelial growth of *Fusarium proliferatum..* It was found that the fermentation solution of *Serratia marcescens*, the dominant strain significantly enriched with calcium at the seedling stage, could substantially inhibit the mycelia growth of *Fusarium solani*. This result was similar to the discovery of Guo et al. The difference was that the inhibition was for different strains of *Fusarium*. Thus, *Serratia marcescens* may have a broad-spectrum antimicrobial effect against *Fusarium* spp.

Peanut southern blight is a widespread disease throughout the world, severely limiting the yield and quality of peanuts. Current research primarily focused on the analysis of the mechanism that caused differences in pathogenicity, determination of fungicide virulence and the screening of antagonistic biocontrol bacteria. Yan et al. [36] conducted a comparative genomic analysis of *Sclerotium rolfsii* with different pathogenicity separately and found that PHI (pathogen-host interaction), CAZymes (carbohydrate active enzymes), effectors and secondary metabolites, etc. might be the main reasons for the difference in pathogenicity. Abo-Zaid et al. [37] used a talc-based medium to ferment *Streptomyces cellulosae* Actino 48 with a high chitinase production which could effectively inhibit the mycelial growth of *Sclerotium rolfsii*. The rhizosphere growth-promoting bacteria *Bacillus pumilus* LX11 [14] and the seed endophytes *Bacillus velezensis* LHSB1 [38] can effectively antagonize *Sclerotium rolfsii* to significantly reduce the incidence of stem rot and can be used as potential biocontrol agents. The present research found that the high concentration fermentation solution of *Novosphingobium panipatense*, a dominant strain significantly enriched by calcium at the pod filling stage, could significantly inhibit the mycelial growth of *Sclerotium rolfsii.* Hence, this strain could be used as a potential biocontrol agent for the southern blight control in the field.

Peanuts are highly susceptible to aflatoxin contamination in the process of planting, harvesting, storage and processing, which seriously affects the food safety and quality of peanuts. It has become a major limiting factor of the peanut industry, as well as an important research area of interest and inquiry for scholars the world over. Zhao et al. [39] identified an important role of chitinase in peanut resistance to *Aspergillus flavus* by differential analysis of transcriptomic and proteomic. Zhao et al. [40] found that miRNAs could regulate peanut resistance to *Aspergillus flavus*. Zhang et al. [41] suggested that non-metabolizable glucose analogs could inhibit the synthesis of aflatoxin and promote *Aspergillus flavus* to produce kojic acid. Meanwhile, Sharma et al. [42] confirmed that pipecolic acid is an important component of peanut seeds that could inhibit the growth of *Aspergillus flavus*. Shakeel et al. [43] demonstrated that culture filtrates and crude extracts of *Streptomyces yanglinensis* 3–10 had significant inhibitory effects on the growth and the aflatoxin production of *Aspergillus flavus*. According to those results, the high concentration of fermentation solution of *Serratia marcescens*, the significant strain in treatment at the seedling stage, could significantly inhibit the mycelial growth of *Aspergillus flavus*. It was inferred that the secondary metabolites of *Serratia marcescens* might contain analogs with chitinase, non-metabolic glucose analogs and pipecolic acid that could inhibit the growth of *Aspergillus flavus* and the production of aflatoxin.

Peanut leaf diseases usually cause early leaf abscission and therefore affect the fullness and yield of pods. Current research has mainly focused on detecting the broad-spectrum fungicides or fungicide combinations, screening antagonistic biocontrol bacteria, localization of QTL, transcriptome analysis [44] and identifying resistance genes etc. [45]. Anco et al. [46] found that the combination of prothion conazole with fluxapyroxad and pyraclostrobin could more effectively prevent and control the black spot of peanut. Shifa et al. [47] sprayed salicylic acid on the leaves to induce systemic acquired resistance in peanuts, significantly reducing the incidence of the late spot. Zhang et al. [48] confirmed that the endophytic fungus *Phomopsis liquidambaris* could simultaneously inhibit the occurrence of the peanut root rot and leaf spot. It was also found that the high concentration culture filtrate of *Novosphingobium panipatense*, a strain enriched in treatmaent at the pod filling stage, could inhibit the growth of the pathogen *Leptosphaerulina arachidicola*. Thus it can be used as a potential biocontrol agent to control the peanut leaf spot in the field.

The rhizosphere is an essential place for plant-microbe interactions. The rhizosphere microbial community plays a crucial role in plant growth and disease defense, which act as the first line of defense for plants against soil-borne diseases. Liu et al. [49] demonstrated that when wheat was attacked by *Fusarium pseudograminearum*, the rhizosphere and intra-root layer were preferentially enriched with *Stenotrophomonas rhizophila* (SR80). SR80 could promote the growth of underground and aboveground parts of the plant, induce systemic acquired resistance and protect the plant from the pathogen. Mendes et al. [50] revealed that the rhizosphere microbial communities of the soybean varieties with high resistance to soil-borne diseases were more complex and more related than those of sensitive varieties. In this study, the number of OTUs specific to the calcium-applied group was higher than that of the non-calcium-applied group. The relative abundance of specific dominant strains was also significantly higher in the group with calcium treatment. The association interactions among the dominant bacterial communities in the calcium-applied group were also significantly more than those in the group without calcium treatment. In the previous field observation, calcium could reduce the incidence of peanut leaf diseases. Comprehensive analysis inferred that calcium could indirectly improve peanut resistance by enriching specific strains to inhibit major pathogens’ growth and enhancing associative interactions between rhizosphere microbial communities.

LEfSe was used to analyze the Biomarkers at different growth stages. *Pseudomonas geniculata* was found to be the Biomarker in the group without calcium application at the seedling stage. *Acinetobacter guillouiae* and *Klebsiella variicola* were the Biomarkers in the group with calcium application. Liu et al. [51] isolated a strain of *Pseudomonas geniculata* BCR5 from rescuegrass and found that the strain promoted germination and seedling growth of aged seeds. *Pseudomonas geniculata* IC-76 isolated from chickpea nodules by Gopalakrishnan et al. [52] significantly increased the number of root nodules, the mass of plant above ground. It also has in vitro plant growth-promoting properties which could be a potential root promoting bacterium. Therefore, it was speculated that *Pseudomonas geniculata* as an important Biomarker in the no-calcium application group at the seedling stage may contribute to seed germination and seedling growth. Cervantes-Vázquez et al. [53] selected *Acinetobacter guillouiae* as a Plant Growth-Promoting Rhizobacteria and studied its effects on the morphophysiology, enzymatic activity and other related effects on greenhouse grown Tomato Saladette seedlings. Wei et al. [54] showed that strain *Klebsiella variicola* DX120 colonised sugarcane was involved in nitrogen fixation and growth promotion. Kim et al. [55] found that *Klebsiella variicola* AY13 strain could produce indole acetic acid (IAA) to promote adventitious root growth in response to flooding stress in soybean. Previous studies have suggested that as important biomarkers in the calcium application group, *Acinetobacter guillouiae* and *Klebsiella variicola* may be potential root growth promoters for peanuts, which are involved in nitrogen fixation and plant growth promotion.

Early studies have focused on how calcium affected peanut embryo development or improved peanut agronomic traits. To investigate the mechanism of how calcium improves peanut’s resistance to disease, the above targeted experiments were designed. This study confirmed that calcium could enhance the interactions of rhizosphere microbial communities. Furthermore, the significant strains enriched with calcium during different growth stages could inhibit the growth of major pathogens at the same stage. Thus, indirectly, the resistance of peanuts was improved. There are still limitations and deficiencies in current research. For example, this study has not yet analyzed what active ingredients in the culture filtrate of the dominant strains enriched with calcium have efficient antibacterial effects. Subsequent studies may provide in-depth analysis of the active components in the fermentation filtrate of the dominant strains that inhibit the growth of pathogens. Even further investigation can be made into the molecular mechanism of how exogenous calcium dramatically enriches specific strains and participates in regulating the synthesis of antibacterial active ingredients.

## Conclusion

These studies have confirmed that calcium could increase the relative abundance of some dominant species. Four dominant strains were enriched with calcium at the seedling stage, such as *Serratia marcescens.* Ten dominant strains were enriched with calcium at the pod filling stage, including *Novosphingobium panipatense. Serratia marcescens* could inhibit the growth of *F. solani* and *A.flavus* at the seedling stage. *Novosphingobium panipatense* could inhibit the growth of *S. rolfsii* and *L. arachidicola* at the pod filling stage. It is hypothesized that calcium could enhance peanut resistance to major soil-borne pathogens by enriching specific dominant strains.

## Methods

### Materials and research site

Xianghua 2008, the calcium-sensitive peanut cultivar were bred by the Dryland Crops Research Institute of Hunan Agricultural University. The acidic and calciprivic soil (pH = 4.61, the pH were 4.64 and 4.77 respectively at the seedling and pod filling stage) was the Quaternary red loam topsoil from Yueguang Ground, Shuyuan Village, Puji Town, Liuyang City, Hunan Province. The exchangeable calcium content was 0.74 cmol (1/2Ca^2+^) kg^− 1^. The test was fulfilled in the Yunyuan practice base of Hunan Agricultural University. The PVC tube with an inner diameter of 36 cm, a height of 75 cm and a wall thickness of 0.5 cm was selected and assembled into a large plastic basin. Each basin contained 90 kg of the test soil. 12.5 g of CaO (with 50 kg plaster (CaO) per 667m^2^) was applied to each basin and mixed with top soil at a depth of 10 cm for the only groups with calcium, while without CaO was the control group. Ten basins of each group were used as replicates.

### Experimental sample collection

The rhizosphere soil is the composite of the root surface soil and the soil surrounding the root. The plants were pulled out respectively at the seedling stage (6.4.2019) and the pod filling stage (8.14.2019). The roots were vigorously shaken to remove the loose bulk soil. Two different sterile scissors were used to cut about 5 cm of the root directly below the rhizome junction. These root tissues were then placed in a 50 ml sterile centrifuge tube containing 40 ml 1xPBS buffer (TransScript # FG701–01) and moderately vortexed for 5minto obtain the root surface microbiota. Centrifugation at 10,000 rpm for 15 min to collect soil sediment in new 10 ml centrifuge tubes (3 repeats in each sample group). MOBIO DNeasy PowerSoil Kit (#12888–50) was used to extract genomic DNA from the rhizosphere microbiome (according to the instructions, each sample was taken 250 mg of soil). NanoDrop2000 instrument was used to detect nucleic acid sample concentration, and then qualified samples were airlifted to Novogene with dry ice (Tianjin, China) for 16S rDNA amplicon sequencing.

### 16S rDNA amplicon sequencing and data statistical analysis

PCR was performed by using diluted genomic DNA as the template. Specific primers 341F/806R were selected according to the sequencing region 16S V3/V4 (Forward primer: 5′-CCTAYGGGRBGCASCAG-3′; Reverse primer: 5′-CCTAYGGGRBGCASCAG-3′). Libraries were constructed with Ion Plus Fragment Library Kit and evaluated for quality by using Qubit@2.0. Afterwards, the quality-checked libraries were sequenced by using Thermofisher’s Ion S5TMXL. To make the result more accurate and reliable, the Raw Data was spliced and filtered to get the Clean Data [56]. All the Clean Reads were clustered, the sequences with ≥97% similarity were assigned as the OTUs [57].

LEfSe analysis was performed (LDA Score filter value defaults to 4) on the OTUs from different groups to screen the dominant strains as key Biomakers in the rhizosphere microbial community. The analysis results are presented as cladogram. Calculated the pearson coefficients of the dominant genera in the rhizosphere with the relative abundance of Top50 and screened the key nodes with relative abundance greater than 0.005%. The valid connections between nodes with pearson correlation coefficient greater than 0.8 and *p* < 0.05 were selected and the node self-connections were filtered out. The interaction network diagrams of the rhizosphere community were drawn respectively in the group without calcium (Fig. 3A), the group with calcium (Fig. 3B), the group at the seedling stage (Fig. 3C) and the group at the pod filling stage (Fig. 3D). The t-test analysis on the relative abundance of all rhizosphere microorganisms in the group with or without calcium application at different growth stages was carried out using SPSS. The results were plotted as histograms using OriginPro2019.

### Dominant strains antagonize Main pathogenic Fungi

*Serratia marcescens*, which significantly enriched by calcium at the seedling stage, was cultured at 30 °C, 180 rpm/h shaker shaking for seven days, centrifuged at 10000 rpm for 15 min, filtered with a 0.22 μm bacterial filter to obtain aseptic fermentation filtrate. The fermentation filtrate mentioned before was diluted in a gradient and mixed with a PDA medium. (2×/fermentation filtrate content 50%, 4×/fermentation filtrate content 25%, 8×/fermentation filtrate content 12.5%, and 16×/fermentation filtrate content 6.25%. × was the dilution multiple). The main pathogens *F. solani* and *A. flavus* isolated from infected peanuts at the seedling stage were inoculated on a PDA plate with gradient mixing of fermentation filtrate for antagonistic confrontation culture. Measure the diameter of the pathogenic fungus colony after seven days, calculate the inhibition rate and linear regression analysis. *Novosphingobium panipatense*, which was significantly enriched by calcium at the pod filling stage, was cultured at 28 °C and 180 rpm/h on a table concentrator for seven days. The bacterial fermentation filtrate was diluted in the same gradient as above. *S. rolfsii* and *L. arachidicola*, the main pathogens isolated from diseased peanut plants at the pod filling stage, were inoculated on a plate with the same method for antagonistic confrontation culture. Measure the diameter of the pathogenic fungus colony after seven days, calculate the inhibition rate and linear regression analysis. The inhibition rate was calculated as [(D_ck_-D_Ca_)/(D_ck_-5)] × 100% (D: colony diameter with units of mm).

## Supplementary Information

**Additional file 1: Subtab 1**. The MCC value of core genera (Top10) from each group. **Subtab 2**. Linear regression analysis of the predominant strains *S. marcescens* and *N. panipatense*, which are significantly enriched by calcium application, against the four main pathogens by gradient dilution of the aseptic fermentation filtrate. **Subfig 1**. Linear regression analysis of the inhibition rate of the dominant bacteria antagonizes the main pathogens. A) Linear regression analysis of the inhibition rate of *S. marcescens* fermentation filtrate against *F. solani* by gradient dilution; B) Linear regression analysis of the inhibition rate of *S. marcescens* fermentation filtrate against *A.flavus* by gradient dilution; C) Linear regression analysis of the inhibition rate of *N. panipatense* fermentation filtrate against *S. rolfsii* by gradient dilution; D) Linear regression analysis of the inhibition rate of *N. panipatense* fermentation filtrate against *L. arachidicola* by gradient dilution.

## Data Availability

The datasets generated and analysed during the current study are available in the NCBI Sequence Read Archive repository under accession number (https://www.ncbi.nlm.nih.gov/bioproject/PRJNA665102/).

## Abbreviations

PGPR: Plant growth promoting rhizobacteria
S.S: The rhizosphere microbiome without calcium application at the seedling stage
SC.S: The rhizosphere microbiome with calcium application at the seedling stage
P.S: The rhizosphere microbiome without calcium application at the pod filling stage
PC.S: The rhizosphere microbiome with calcium application at the pod filling stage
OTUs: Operational taxonomic units
LEfSe: LDA Effect Size
LDA: Linear discriminant analysis
