# Supplementary material for: The resistance of peanut to soil-borne pathogens improved by rhizosphere probiotics under calcium treatment
Source: BMC Microbiol. 2021 Oct 29;21:299. doi: 10.1186/s12866-021-02355-3 (PMC8555263; doi:10.1186/s12866-021-02355-3)
Supplement: Supplementary file 1 — Additional file 1: Subtab 1. The MCC value of core genera (Top10) from each group. Subtab 2. Linear regression analysis of the predominant strains S. marcescens and N. panipatense, which are significantly enriched by calcium application, against the four main pathogens by gradient dilution of the aseptic fermentation filtrate. Subfig 1. Linear regression analysis of the inhibition rate of the dominant bacteria antagonizes the main pathogens. A) Linear regression analysis of the inhibition rate of S. marcescens fermentation filtrate against F. solani by gradient dilution; B) Linear regression analysis of the inhibition rate of S. marcescens fermentation filtrate against A.flavus by gradient dilution; C) Linear regression analysis of the inhibition rate of N. panipatense fermentation filtrate against S. rolfsii by gradient dilution; D) Linear regression analysis of the inhibition rate of N. panipatense fermentation filtrate against L. arachidicola by gradient dilution. [file 12866_2021_2355_MOESM1_ESM.docx]

**Supplementary material**

Subtab.1 The MCC value of core genera (Top10) from each group

| CK | | C | | S | | P | |
| --- | --- | --- | --- | --- | --- | --- | --- |
| Taxonomy | MCC | Taxonomy | MCC | Taxonomy | MCC | Taxonomy | MCC |
| *Phenylobacterium* | 85800 | *Cupriavidus* | 6480 | *Candidatus Solibacter* | 1134 | *Cupriavidus* | 81528 |
| *Methylobacterium* | 85800 | *Gemmatimonas* | 6480 | *Herbaspirillum* | 1134 | *Gemmatimonas* | 81480 |
| *Sphingomonas* | 85800 | *Bryobacter* | 6480 | *Klebsiella* | 1128 | *Bryobacter* | 81480 |
| *Paenibacillus* | 85800 | *Enterobacter* | 6480 | *Dyella* | 986 | *Enterobacter* | 81480 |
| *Ralstonia* | 85722 | *Brevibacillus* | 5760 | *Pandoraea* | 864 | *Brevibacillus* | 81480 |
| *Elizabethkingia* | 80670 | *Dyella* | 5046 | *Chujaibacter* | 846 | *Dyella* | 80809 |
| *Delftia* | 80670 | *Sphingomonas* | 5046 | *Bryobacter* | 846 | *Sphingomonas* | 80642 |
| *Lysinibacillus* | 45510 | *Pseudomonas* | 5046 | *Bradyrhizobium* | 290 | *Pseudomonas* | 80642 |
| *Candidatus_Solibacter* | 40358 | *Candidatus_Solibacter* | 738 | *Bacillus* | 152 | *Candidatus_Solibacter* | 41342 |
| *Phenylobacterium* | 40358 | *Cupriavidus* | 738 | *Sphingomonas* | 146 | *Mycobacterium* | 40345 |

CK: The samples from the control group without calcium treatment

C: The samples from the group with calcium treatment

S: The samples from the group at the seedling stage

P: The samples from the group at the podding stage

Subtab.2 Linear regression analysis of the predominant strains *S. marcescens* and *N. panipatense*, which are significantly enriched by calcium application,

against the four main pathogens by gradient dilution of the aseptic fermentation filtrate

| Antagonistic bacteria  vs Pathogenic fungi |  | Inhibition rate in different proportion of the filtrated fermentation liquid | | | | | Linear regression equation |
| --- | --- | --- | --- | --- | --- | --- | --- |
|  |  | CK | 50% | 25% | 12.5% | 6.25% |  |
| *S. marcescens* vs *F. solani* | Colony diameter (cm) | 6.45±0.05 | 1.12±0.10 | 3.45±0.05 | 5.50±0.28 | 6.22±0.16 | y = -26.899x + 104.13  R^2^ = 0.955 |
|  | Inhibition rate (%) |  | 82.69 | 46.51 | 14.73 | 3.62 |  |
| *S. marcescens* vs *A.flavus* | Colony diameter (cm) | 6.12±0.12 | 0.68±0.06 | 2.90±0.13 | 4.38±0.15 | 5.90±0.05 | y = -28.011x + 113.35  R^2^ = 0.9898 |
|  | Inhibition rate (%) |  | 88.83 | 52.59 | 28.34 | 3.54 |  |
| *N. Panipatense* vs *S. rolfsii* | Colony diameter (cm) | 6.75±0.20 | 0.55±0.05 | 2.90±0.13 | 4.50±0.13 | 5.95±0.27 | y = -26.37x + 114.44  R^2^ = 0.9863 |
|  | Inhibition rate (%) |  | 91.85 | 57.04 | 33.33 | 11.85 |  |
| *N. panipatense* vs *L. arachidicola* | Colony diameter (cm) | 6.28±0.10 | 0.58±0.03 | 2.93±0.10 | 4.75±0.10 | 5.35±0.10 | y = -25.65x + 109.95  R^2^ = 0.9427 |
|  | Inhibition rate (%) |  | 90.72 | 53.32 | 24.40 | 14.85 |  |

Note: Values are mean±standard error of three replicate

Subfig.1

**
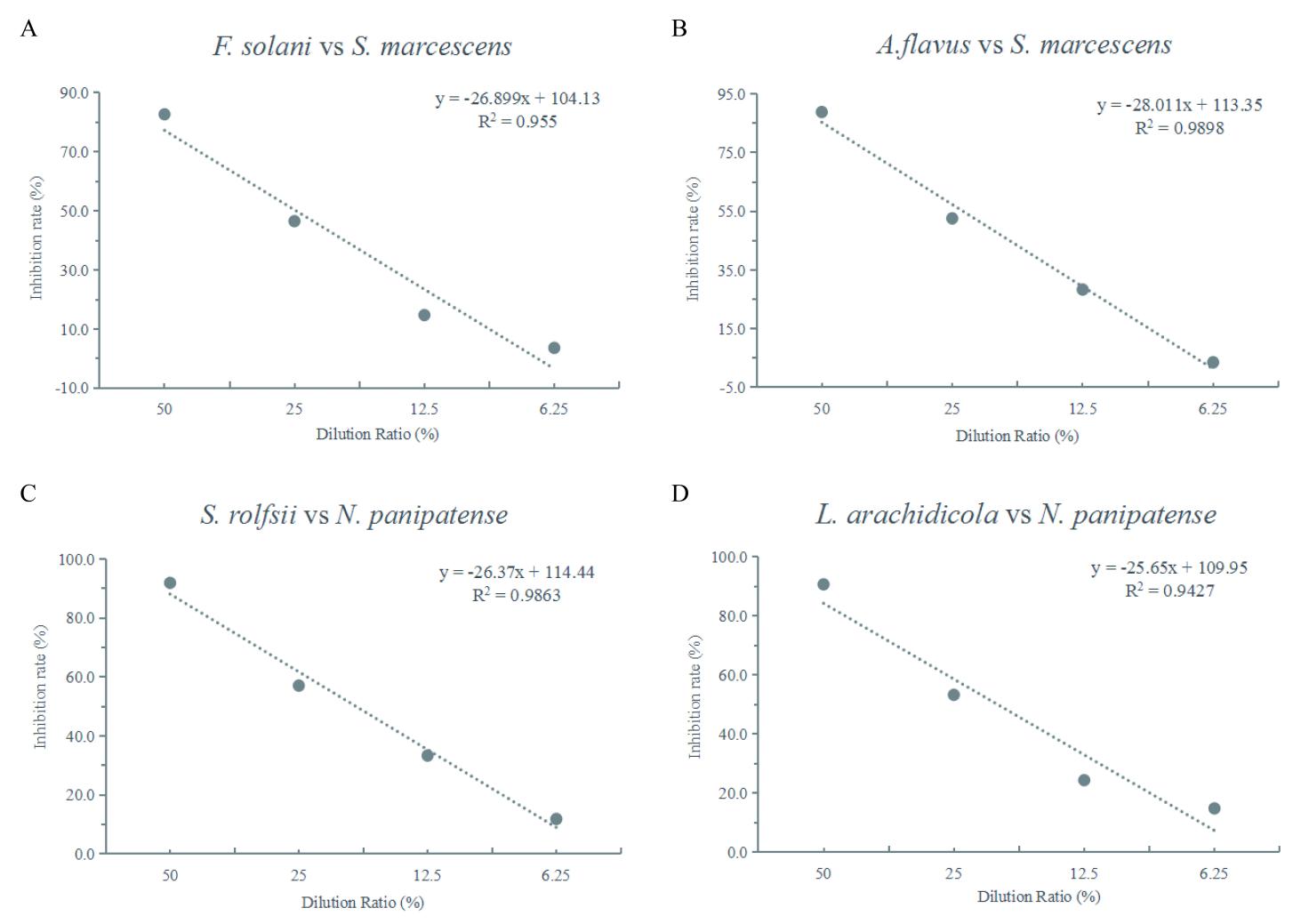
**

Subfig.1 Linear regression analysis of the inhibition rate of the dominant bacteria antagonizes the main pathogens. A) Linear regression analysis of the inhibition rate of *S. marcescens* fermentation filtrate against *F. solani* by gradient dilution; B) Linear regression analysis of the inhibition rate of *S. marcescens* fermentation filtrate against *A.flavus* by gradient dilution; C) Linear regression analysis of the inhibition rate of *N. panipatense* fermentation filtrate against *S. rolfsii* by gradient dilution; D) Linear regression analysis of the inhibition rate of *N. panipatense* fermentation filtrate against *L. arachidicola* by gradient dilution.
